# A Mutation in *ZNF143* as a Novel Candidate Gene for Endothelial Corneal Dystrophy

**DOI:** 10.3390/jcm8081174

**Published:** 2019-08-06

**Authors:** Yonggoo Kim, Hye Jin You, Shin Hae Park, Man Soo Kim, Hyojin Chae, Joonhong Park, Dong Wook Jekarl, Jiyeon Kim, Ahlm Kwon, Hayoung Choi, Yeojae Kim, A Rome Paek, Ahwon Lee, Jung Min Kim, Seon Young Park, Yonghwan Kim, Keehyoung Joo, Jooyoung Lee, Jongsun Jung, So-Hyang Chung, Jee Won Mok, Myungshin Kim

**Affiliations:** 1 Department of Laboratory Medicine, College of Medicine, The Catholic University of Korea, Seoul 06591, Korea; 2 Catholic Genetic Laboratory Center, Seoul St. Mary’s Hospital, College of Medicine, The Catholic University of Korea, Seoul 06591, Korea; 3 Cancer Cell and Molecular Biology Branch, Division of Cancer Biology, National Cancer Center, Gyeonggi-do 10408, Korea; 4 Department of Ophthalmology and Visual Science, Seoul St. Mary’s Hospital, College of Medicine, The Catholic University of Korea, Seoul 06591, Korea; 5 Department of Hospital Pathology, College of Medicine, The Catholic University of Korea, Seoul 06591, Korea; 6 Genoplan Korea, Inc., Seoul 06221, Korea; 7 Department of Life Systems, Sookmyung Women’s University, Seoul 04312, Korea; 8 Center for in Silico Protein Science, Korea Institute for Advanced Study, Seoul 02455, Korea; 9 Center for Advanced Computation, Korea Institute for Advanced Study, Seoul 02455, Korea; 10 School of Computational Sciences, Korea Institute for Advanced Study, Seoul 02455, Korea; 11 Syntekabio Inc., Daejeon 34025, Korea; 12 Catholic Institutes of Visual Science, The Catholic University of Korea, Seoul 06591, Korea

**Keywords:** *ZNF143* gene, endothelial corneal dystrophy, posterior polymorphous corneal dystrophy (PPCD), novel mutation, reverse epithelial-to-mesenchymal transition (reverse EMT), three-dimensional modeling, array-comparative genomic hybridization (CGH), whole exome sequencing (WES), transfection

## Abstract

Corneal dystrophies (CDs) are a diverse group of inherited disorders with a heterogeneous genetic background. Here, we report the identification of a novel *ZNF143* heterozygous missense mutation in three individuals of the same family with clinical and pathological features that are consistent with endothelial CD. Ophthalmologic examination revealed diffuse corneal clouding and edema with decreased endothelial cell density. Pathological findings showed increased corneal thickness due to edema of basal epithelial cells and stroma, and abnormal metaplastic endothelium with stratified epithelium-like changes. Patients’ metaplastic corneal endothelial cells expressed predominantly cytokerain 7, cytokeratin 19, and E-cadherin. Although Sanger sequencing did not detect any mutation associated with endothelial CDs, whole exome sequencing identified the *ZNF143* c.937G>C p.(Asp313His) mutation as a candidate gene for our patients’ endothelial CD. In-vitro functional studies demonstrated that mutant *ZNF143* promoted the mesenchymal-to-epithelial transition; it upregulated the expression of genes associated with epithelialization in human corneal endothelial cells. Additionally, proinflammatory cytokine responsive genes were significantly enriched after mutant *ZNF143* transfection, which may contribute to the severe phenotype of the three patients. These findings link a mutation in *ZNF143* with endothelial CD for the first time.

## 1. Introduction

Corneal dystrophies (CDs) are a diverse group of inherited disorders with a heterogeneous genetic background. Patients with various CDs are diagnosed according to the International Committee for Classification of Corneal Dystrophies (IC3D) guidelines through ophthalmologic examination, pathologic examination, and genetic analysis [1]. Posterior polymorphous corneal dystrophy (PPCD) is a rare, autosomal dominantly inherited, corneal endothelial dystrophy characterized by an abnormal transition of the corneal endothelium into stratified squamous epithelium and corneal edema [2,3]. PPCD is diagnosed based on ophthalmologic findings, but molecular genetic analysis can differentiate various clinically similar CDs [4,5]. Locus heterogeneity has been identified for PPCD with mutations in the *COL8A2* (collagen type VIII alpha 2 chain) gene on chromosome 1p34.3 (PPCD2), the *ZEB1* (zinc finger E-box binding homeobox 1) gene on chromosome 10p11.2 (PPCD3), and the *GRHL2* (grainyhead like transcription factor 2) gene on chromosome 8q22.3 (PPCD4) [6,7,8,9]. A locus for PPCD1 maps to the pericentromeric region of chromosome 20. The phentotypes described as PPCD share similarities with a severe phenotype of CD that was originally termed congenital hereditary endothelial dystrophy (CHED). Patients with autosomal dominant CHED1 showed evident corneal opacity by 1 year of age and required corneal transplantation [10]. Previous studies demonstrated that these two disorders could be caused by mutations within the same gene but displayed variable phenotypic severity. This region was recently implicated in the transcriptional dysregulation of the *OVOL2* (ovo like zinc finger 2) gene on chromosome 20p11.23 [11]. Therefore, CHED1 is no longer classified as a distinct disease entity but is considered a more severe form of PPCD1 in the updated 2015 IC3D. CHED2 is an autosomal recessive disease and presented with corneal opacity at birth or in the immediate newborn period. Mutations in *SLC4A11* (solute carrier family 4 member 11), a transmembrane protein in the family of sbicarbonate transporters, are present in the majority of CHED2 patients [12]. However, not all genetic causes of endothelial CDs are known, thus, it is possible to identify novel candidate genes for CDs via continuous studies. Here, we report the identification of a novel heterozygous missense mutation in *ZNF143* (zinc finger protein 143) found in three individuals of the same family with endothelial CD.

## 2. Experimental Section

### 2.1. Ethics Statement

All individuals including family members were investigated after obtaining informed consent in accordance with the Declaration of Helsinki. In addition, the study was approved by the Seoul St. Mary’s Hospital institutional ethics committee (KC14RISI0419, approved on 2 June 2014).

### 2.2. Subject and Ophthalmologic Examination

The medical records of the three patients were investigated; the age, gender, visual acuity, family history, and previous medical records of the patients were reviewed. Ocular examinations were performed, including measurements of the best-corrected visual acuity (BCVA), intraocular pressure (IOP), central corneal thickness, and slit lamp biomicroscopy of the anterior segment and fundus. Visual activity was measured based on a vision chart using the Logarithm of the Minimum Angle of Resolution (logMAR scale; JV institute, Seoul, Korea) according to International Organization for Standardization 8596. A non-contact Konan specular microscope (RoboCa; Konan, Hyogo, Japan) was used to evaluate the corneal endothelium.

### 2.3. Sanger Sequencing of OVOL2, COL8A2, ZEB1, SLC4A11 and GRHL2

Genomic DNA was isolated from the peripheral blood leukocytes using the QIAmp DNA Mini Kit (Qiagen, Hamburg, Germany). Polymerase chain reaction (PCR) was carried out using the previously published primer sets for *COL8A2*, *ZEB1*, and *SLC4A11* [6,7,13]. Primers for *OVOL2* and *GRHL2* were designed by the authors (Table 1). All of the coding exons, flanking intron/exon boundaries, and promotor regions for the relevant genes were amplified. PCR amplicons were bi-directionally sequenced using the Big Dye terminator v3.1 cycle sequencing kit (Applied Biosystems, Foster City, CA, USA) on an ABI PRISM 3100 Genetic Analyzer (Applied Biosystems). The chromatograms were analyzed with the Sequencher software version 5.0 (Gene Codes, Ann Arbor, MI, USA). Sequence variants were confirmed by sequencing two or more independent PCR reactions. The GenBank accession numbers were NG_046859.1 and NM_021220.3 for *OVOL2,* NG_016245.2 and NM_005202.2 for *COL8A2*, NG_017048.1 and NM_030751.5 for *ZEB1*, NG_017072.1 and NM_032034.3 for *SLC4A11*, and NG_011971.2 and NM_024915.3 for *GRHL2*.

### 2.4. Array-Comparative Genomic Hybridization (CGH)

Array-CGH analysis was performed to detect the genomic rearrangements or deletions that may have occurred in the known CD-associated gene regions. A whole genome 720K NimbleGen CGX cytogenetic array chip (Roche NimbleGen, Inc., Madison, WI, USA), which includes 719,336 oligonucleotide probes per haploid genome, was used. For array-CGH analysis, 500 ng of test DNA and sex-matched reference DNA (Promega, Napeen, ON, Canada) were random-prime labeled with Cy3 and Cy5, respectively, using the NimbleGen labeling kit. The samples were hybridized to the array at 42 °C, then washed and scanned using the NimbleGen MS 200 Microarray Scanner. The data were analyzed using DEVA version 1.0.2 software (Roche NimbleGen, Inc., Madison, WI, USA).

### 2.5. Haplotype Analysis

Haplotype analysis was performed with the aim of refining the previously identified region of linkage. Genotyping analysis was performed using eight polymorphic microsatellite markers, including D20S98, D20S114, D20S48, D20S182, M189K21, D20S139, D20S106, and D20S107 on chromosome 20p12.1–20q12, which were fluorescently labeled and amplified by PCR [14]. The fluorescent PCR products were separated against the Genescan-500LIZ size standard on an ABI 3130XL Genetic Analyzer (Applied Biosystems, Foster City, CA, USA), and alleles were assigned using the GeneMapper software, version. 4.1 (Applied Biosystems, Foster City, CA, USA).

### 2.6. Whole Exome Sequencing

We enrolled patients for whole exome sequencing to identify genetic variants potentially associated with the disease [15]. Family pedigrees suggested autosomal dominant inheritance with complete penetrance. We obtained whole exome sequencing data for three patients (II-3, III-1 and III-2) and two unaffected family members (I-2 and II-6) and carried out analysis to identify shared variants. Genomic DNA was used for library preparation, which included the shearing and ligation of sequencing adaptors. Target enrichment of the samples was performed with the SureSelect Human Exome Kit V.4 (Agilent Technologies, Santa Clara, CA, USA). The exome DNA was captured using a SureSelect All Exon V4+UTRs kit (Agilent Technologies) and sequenced as paired-end 150 bp reads on the HiSeq 2500 platform (Illumina, San Diego, CA, USA). The obtained sequence reads were aligned to the human reference genome sequence 19 (hg19) using Novoalign v3.01.01 (Novacraft, Selangor, Malaysia). The BAM files were then processed by base quality recalibration, duplicate removal, and local realignment following the GATK’s best practice workflow for variant calling. After filtering steps based on segregation status, variants co-segregating with the disease remained. We reviewed all identified variants, including intronic variants and synonymous exonic variants. To define the novel variants, we searched public databases of genetic variants including the Human Gene Mutation Database (HGMD; http://www.hgmd.cf.ac.uk/ac/index.php); the Genome Aggregation Database (gnomAD; https://gnomad.broadinstitute.org/); the Single Nuclotide Polymorphism Database (dbSNP; https://www.ncbi.nlm.nih.gov/snp/); the International Genome Sample Resource (IGSR; http://www.internationalgenome.org/) of the 1000 Genomes project; and the National Heart, Lung, and Blood institute Grand Opportunity Exome Sequencing Project (NHLBI GO-ESP; https://esp.gs.washington.edu/drupal/), as well as an ethnic-specific Korean genome database comprising 1244 alleles: the Korean Reference Genome Database (KRGDB; http://152.99.75.168/KRGDB/). When a novel missense variant was identified, in-silico analyses were performed using the Sorting Intolerant From Tolerant (SIFT; http://sift.jcvi.org), the Polymorphism Phenotyping v2 (Polyphen-2; http://genetics.bwh.harvard.edu/pph2), and the Mutation Taster (http://www.mutationtaster.org) to assess if the substitutions were predicted as potentially pathogenic. Also, in the case of a missense variant, the evolutionary conservations of the involved amino acids were investigated using the resources at the Evola website (http://www.h-invitational.jp). We further classified variants in candidate genes according to the American College of Medical Genetics and Genomics and the association for Molecular Pathology (ACMG/AMP) guidelines for variant interpretation in Mendelian disorders [16], and all variants were scored and classified into five pathogenicity groups (class 1: benign; class 2: likely benign; class 3: uncertain significance (VUS); class 4: likely pathogenic; class 5: pathogenic). While these guidelines were developed for the interpretation of variants observed in a clinical diagnostic laboratory setting and were not intended to address the pathogenicity of variants in genes not yet established to be disease-causing, we have categorized all variants in novel candidate genes based on available evidence for variant pathogenicity, as appropriate.

### 2.7. Modeling of ZNF143 p.Asp313His

The protein segment ranging from residue numbers 237 to 440 contains seven zinc-fingers, each consisting of an alpha helix and an anti-parallel beta sheet, and binds to DNA molecules. To generate a three-dimensional (3D) model of ZNF143 p.Asp313His, we employed a recently proposed template-based modeling method [17]. The method uses global optimization to generate protein 3D models and was shown to be quite successful in recent Critical Assessment of Protein Structure (CASP) protein structure prediction experiments (CASP7 to CASP12) [18]. Especially, in CASP11, it was ranked as the best method in the category of template-based modeling. The protocol identified multiple templates including 2I13, 1UBD and 2GLI. To build the complex structure of the protein with a DNA molecule, the orientation of the DNA molecule in 2I13 was utilized. Using ZNF143 p.Asp313His and the three templates, multiple sequence alignment trials were carried out using Multiple Sequence Alignment by Conformational Space Annealing (MSACSA) [19], and the sequence identities between the target and the templates were about 40% including loop regions. Using the resultant alignment and three templates, single-chain models were generated by using the global optimization method, conformational space annealing [20,21]. The complex structure was subsequently minimized and equilibrated by the AMBER program [22] using an ff14SB force field with the TIP3P explicit water model. Zinc binding was modeled using the Zinc AMBER Force Field (ZAFF) [23]. The complex structures of ZNF143 p.Asp313His and DNA were generated using the UCSF Chimera [24].

### 2.8. In Vitro Functional Studies of ZNF143 p.Asp313His

#### 2.8.1. Isolation and Culture of Human Corneal Endothelial Cells (hCECs)

Corneal explants were washed three times with ice-cold phosphate-buffered saline (PBS) containing penicillin-streptomycin (100 U/mL) and gentamicin (50 μg/mL). Descemet membranes containing the hCECs were stripped from donor corneas and digested with 1 mg/mL collagenase A (Roche Applied Science, Penzberg, Germany) at 37 °C for 1 h. The cells were then centrifuged (300× *g* for 5 min), and the primary cells were resuspended in EMB™-2 basal medium (Lonza, Basal, Switzerland). All maintenance media were supplemented with growth supplement (Lonza); B27 (Gibco); and Rho-associate, coiled-coil containing protein kinase (ROCK) inhibitor (Y27632). All cells were grown at 37 °C in a humidified 5% CO_2_ atmosphere.

#### 2.8.2. siRNA-Mediated Silencing of Human ZNF143

*ZNF143* affects the expression of epithelial marker proteins, such as E-cadherin, by regulating *ZEB1* expression in colon cancer cells [25]. Thus, we investigated whether the ZNF143 p.Asp313His mutation is involved in endothelial CD-causing gene expression through *ZEB1*, in primary cultured hCECs, the colon cancer cell line HCT116, human embryonic kidney cells HEK293, and dermal microvascular endothelial cells HMEC-1 (ATCC^®^ CRL-3243™; ATCC, Manassas, VA, USA). To reduce *ZNF143* expression in cells, cells (7 × 10^4^/well) were grown in 12-well plates for 24 h, and the control (siControl) and *ZNF143* (siZNF143; Santa Cruz Biotechnology Inc., Santa Cruz, CA, USA) were transfected with Lipofectamine 2000 (Invitrogen, Carlsbad, CA, USA) following the manufacturer’s instructions and incubated for an additional 24 h (HCT116, HEK293) or 48 h (hCECs, HMEC-1).

#### 2.8.3. Transfection of Human ZNF143 (Wild Type and Mutant)

*ZFN143* cloning in vector pcDNA3.1(+) was prepared as follows. PCR for the cloning of wild and mutant human *ZNF143* was performed. A *ZNF143* fragment was purified using the Labopass gel extraction kit (Cosmogenetech, Seoul, Korea) according to the manufacturer’s instructions. A pcDNA3.1(+) plasmid was digested with NheI and XbaI (New England Biolabs Ltd., Hitchin, UK). After digestion, *ZNF143* fragments were inserted into pcDNA3.1(+) vector and the following ligation was performed using T4 DNA ligase (New England Biolabs Ltd.). Transformed *Escherichia coli* were selected using Luria-Bertani (LB) agar containing ampicillin. The recombinant vector was extracted using a Labopass genomic DNA isolation kit (Cosmogenetech). To further confirm the role of ZNF143, cells were transfected with plasmids encoding ZNF143 wild type, mutant, a full-length tagged with FLAG, or an empty vector via Lipofectamine 2000. The cells were grown for 24–48 h and harvested for further study.

#### 2.8.4. Gene Expression Analysis

Cells were employed in a variety of experiments including microarray, reverse transcription PCR (RT-PCR), RT-quantitative PCR (RT-qPCR), and immunoblotting analysis. Total cellular RNA was extracted with RNeasy kit (Qiagen, Valencia, CA, USA) and was dissolved in RNase-free water. One color microarray-based gene expression analysis was performed to identify the changes of the gene expression profile of the *ZNF143* mutation. Gene expression was examined using a SurePrint G3 Human GE 8 × 60K V2 Microarray Kit (Agilent Technologies) according to the manufacturer’s instructions. The microarray was scanned in a high-resolution microarray scanner (Agilent Technologies). The average fluorescence intensity for each spot was calculated, and the local background was subtracted with the Agilent Feature Extraction software package. All data normalization and selection of up- and down-regulated genes was performed using GeneSpring GX 7.3 (Agilent Technologies). The averages of normalized ratio were calculated by dividing the average of the test channel signal intensity by the control channel signal intensity. Genes that changed more than 4-fold in mutant *ZNF143* transfected corneal endothelial cells versus control transfected cells were used as the testing gene set. A gene network analysis was performed for the representation of relationships among biomolecules using the Ingenuity^®^ Pathway Analysis (IPA; Qiagen) [26]. Additionally, gene set enrichment analysis (GSEA) was used to identify a gene set collection of biological processes [27]. GSEA was run using 1000 permutations of gene sets in the website gene matrix database with the weighted enrichment statistic.

For RT-PCR, RNA samples (1 μg) were reverse-transcribed at 50 °C for 60 min in 20 μL buffer (10 mM Tris; pH 8.3; 50 mM KCl; 5 mM MgCl_2_; and 2.5 μM each of dATP, dCTP, dGTP, and dTTP) in the presence of oligo (dT) primer. Hot-start PCR was used to increase the specificity of amplification. The PCR products were subjected to electrophoresis on 1.5% (wt/vol) agarose gels, and the resulting bands were visualized with ethidium bromide and photographed using the GelDoc program (Bio-Rad, Hercules, CA, USA). Some primer sets *KRT7* (keratin 7), *KRT19* (keratin 19)*, CDH1* (cadherin 1), and *GAPDH* (glyceraldehyde-3-phosphate dehydrogenase) were also used for RT-qPCR to confirm Taqman PCR results by using a LightCycler^®^ 480 SYBR Green I Master (Roche Diagnostics Corp., Indianapolis, IN, USA) following the manufacturer’s instructions with a varied amount of template cDNA. We also measured the expression of four PPCD-associated genes by RT-qPCR: *ZNF143* (Hs00366181_m1), *COL8A2* (Hs00697025_m1), *ZEB1* (Hs00232783_m1), and *OVOL2* (Hs00221902_m1). RT-qPCR was performed using the TaqMan^®^ gene expression assay (Applied Biosystems, Foster City, CA, USA) according to the manufacturer’s instructions. All reactions were performed using the ABI ViiA7^TM^ Real-Time PCR system (Applied Biosystems, Foster City, CA, USA). PCR conditions were 10 min at 95 °C, and 40 cycles of 15 s at 95 °C and 1 min at 60 °C. Gene expression levels were estimated in triplicate by the 2^−ΔΔCt^ method with human *GAPDH* (Hs99999905_m1) level as a reference gene.

We conducted a luciferase reporter assay to test wild-type or mutant human *ZNF143* regulated *ZEB1* expression at the transcription level. Twenty-four and 48 h post-transfection, HEK293 cells were lyzed, and the luciferase activities were measured with the Dual-Luciferase^®^ Reporter Assay System (Promega, Madison, WI, USA) according to the manufacturer’s instructions.

#### 2.8.5. Immunoblotting

Mouse monoclonal antibodies against β-actin and ZNF143 were obtained from Santa Cruz Biotechnology Inc. Rabbit polyclonal antibodies against E-cadherin, N-cadherin, α-smooth muscle actin (α-SMA), snail, and α-tubulin were purchased from Cell Signaling Technology Inc. (Beverly, MA, USA). Mouse monoclonal antibodies against cytokeratin 7 (CK7) were purchased from Thermo Fisher Scientific Inc. (Rockford, IL, USA). A rabbit polyclonal antibody against ZEB1 was purchased from Sigma-Aldrich Corporation (St. Louis, MO, USA). Horseradish peroxidase-conjugated anti-mouse and anti-rabbit antibodies were purchased from Cell Signaling Technology Inc. (Beverly, MA, USA). Protein samples were heated to 95 °C for 7 min and subjected to sodium dodecyl sulfate–polyacrylamide gel electrophoresis on 6% or 8% acrylamide gels. They were then transferred to polyvinylidene difluoride membranes for 1 h at 350 mA with a Bio-Rad transfer unit. The membranes were blocked for 30 min in Tris-buffered saline with 0.15% Tween 20 (TBST) with 5% nonfat-dried milk. After which, they were incubated over-night at 4 °C with the primary antibody in TBST and 2% nonfat-dried milk, followed by 1 h with horseradish peroxidase-conjugated anti-mouse or rabbit antibody. The blots were developed with an enhanced chemiluminescence kit (West-ZOL^®^ plus, Western Blot Detection System; Intron Biotechnology Inc., Daejeon, Korea), and the quantification of band intensity on XAR-5 film (Eastman Kodak Co., Rochester, NY, USA) was measured by the Quantity One software (Bio-Rad).

### 2.9. Immunohistochemical Staining

Slides from each patient and controls were stained with hematoxylin and eosin for the morphological assessment of the endothelial layer by light microscopy. Immunohistochemistry for CK7 (clone OV-TL12/30, M7018, 1:50, DAKO, Carpinteria, CA, USA), CK19 (clone RCK108, M0888, 1:100, DAKO), E-cadherin (clone 4A2C7, #33-4000, 1:200, Zymed, San Francisco, CA, USA), ZEB1 (clone 1H1F1, 66279-1-Ig, 1:200, proteintech, Carpinteria, IL, USA), COL8A2 (aa571-599, LS-B13195-200, 1:25, Lifespan bioSciences, Seattle, WA, USA) and ZNF143 (L-26, sc-100983, 1:100, Santa Cruz Biotechnology, Dallas, Texas, UT, USA) was performed using a Dako Omnis automatic immunohistochemical staining system (Agilent).

## 3. Results

### 3.1. Patients Characteristics

**Figure 1:**
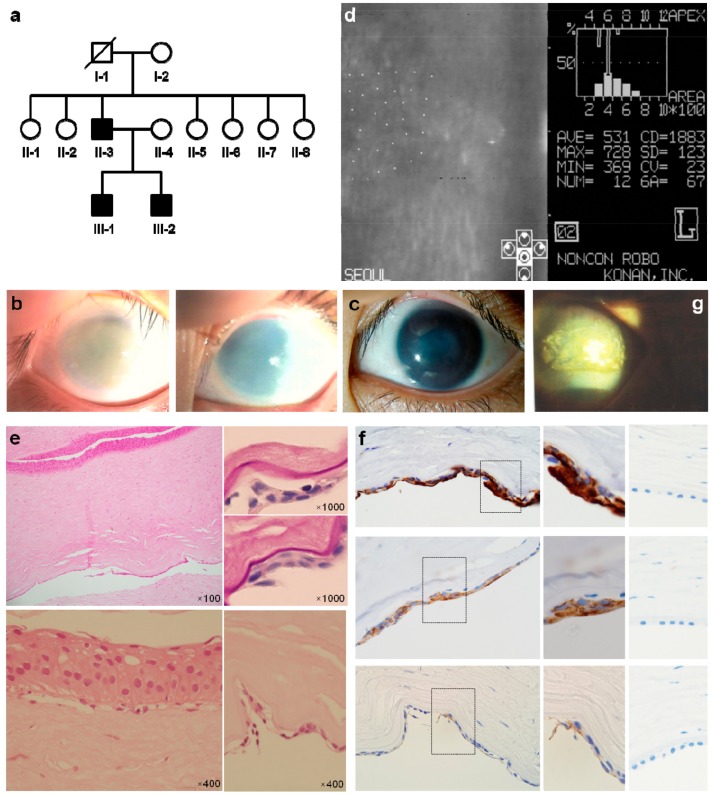
Clinical, ophthalmological and pathological characteristics. (**a**) Pedigree of affected family showing autosomal dominant inheritance. (**b**) Slit-lamp photograph of an 8-year-old boy (III-1) showing bilateral diffuse-marked corneal edema (left: left eye, right: right eye). The corneal diameter was not enlarged and no other corneal abnormalities, such as Haab’s striae or endothelial vesicles and lines, were observed. (**c**) Slit-lamp photograph of his younger brother (III-2) at the age 18. Corneal edema with a diffuse ground glass appearance was noticed in his left eye. There was no corneal vascularization. Descemet’s membrane appeared thickened. (**d**) The central corneal thickness (III-2) was 861 μm and the endothelial cell density was 1883 cells/mm^2^. (**e**) The cornea (III-2) shows increased thickness due to edema of basal epithelial cells and stroma, and abnormal metaplastic endothelium with stratified epithelium-like changes. Hematoxylin and eosin staining and Periodic acid-Schiff staining. (**f**) Patient’s metaplastic corneal endothelial cells express predominantly cytokerain 7 (CK7) (upper), CK19 (middle), and E-cadherin (lower) while they were not expressed in control corneal endothelial cells (right). (**g**) Slit-lamp photograph of patients’ father (II-3) at the age of 45 showed spheroidal corneal degeneration with neovascularization.

An 8-year-old male (III-1) visited our clinic due to the corneal clouding of both eyes, which had developed from birth and progressed slowly thereafter (Figure 1a). Their best-corrected vision was 2/100 in both eyes with pendular nystagmus. Bilateral marked corneal thickening extending to the limbus was a characteristic found by slit lamp biomicroscopic examination (Figure 1b). It was impossible to evaluate corneal endothelium by specular microscopy and measure the central corneal thickness due to severe corneal edema. The patient’s corneal diameter was not enlarged, and no other corneal abnormalities, such as Haab’s striae or endothelial vesicles and lines, were detected.

The axial length was 23.61 mm in the right eye and 22.54 mm in the left eye. The intraocular pressure was 16 mmHg in the right eye and 18 mmHg in the left eye. Penetrating keratoplasty performed in both eyes at 9 years of age failed to gain transparency of the cornea. Bilateral penetrating keratoplasty specimens showed severe epithelial intercellular edema with cyst formation.

His younger brother (III-2) visited our hospital at 9 years of age due to corneal clouding in both eyes. The best-corrected visual acuity was 20/60 in the right eye and 20/100 in the left eye. The central corneal thickness measured by ultrasound pachymetry was 922 μm and 990 μm in the right and left corneas, respectively. He received a penetrating keratoplasty twice in his right eye, but the grafted cornea re-opacified. During the subsequent 9 years, the corneal clouding of his left eye did not progress, and the best-corrected vision remained at 20/50 (Figure 1c). At 18 years of age, the central corneal thickness was 861 μm, and specular microscopy revealed an endothelial cell density of 1883 cells/mm^2^ in his left eye (Figure 1d). The right cornea showed increased thickness due to edema of basal epithelial cells and stroma, and an abnormal metaplastic endothelium with stratified epithelium-like changes (Figure 1e). We evaluated the protein expression in patients’ corneal tissues by immunohistochemical staining. The metaplastic endothelial cells were diffusely positive for (cytokeratin 7) CK7 and CK19, and focally positive for E-cadherin by immunohistochemical staining (Figure 1f). These epithelial-like metaplastic changes of corneal endothelium are known to be the characteristic pathological findings of PPCD. Their father (II-3) had received a keratoplasty at 9 years of age because of severe corneal edema and now displayed spheroidal corneal degeneration with neovascularization (Figure 1g).

### 3.2. ZNF143 c.937G>C p.(Asp313His) Mutation Was Identified as a New Candidate for Endothelial CD

In an attempt to determine causative mutations for the patients, we first carried out Sanger sequencing of endothelial CD-associated genes (*COL8A2*, *ZEB1*, *SLC4A11*, and *OVOL2*) and performed array-comparative genomic hybridization analysis [8] on the three affected patients. No mutations were identified in these CD-associated genes (Figure S1a). Haplotype analysis using eight polymorphic microsatellite markers on chromosome 20p12.1–20q12 did not reveal a common shared haplotype, which suggested that the causal mutation is not located on the pericentric region of chromosome 20 (Figure S1b) [14]. Next, we performed whole exome sequencing (WES) [28] for the three patients and two unaffected family members (I-2 and II-6). Given the apparent autosomal dominant inheritance pattern of disease within the family, we assumed that the causal mutation would be present in the heterozygous state. WES revealed 10 potentially causal sequence variants: *TXNIP* (thioredoxin interacting protein), *EPC2* (enhancer of polycomb homolog 2), *KCNV2* (potassium voltage-gated channel modifier subfamily V member 2), *NUTM2F* (NUT family member 2F), *AOPEP* (aminopeptidase O), *TNC* (tenascin C), *RABGAP1* (RAB GTPase activating protein 1), *ZNF143*, *ERICH6B* (glutamate rich 6B), and *MZF1* (myeloid zinc finger 1). Among them, *ZNF143* represented the best candidate for endothelial CD in the three patients. Supporting evidence used for classification of pathogenicity of variants in candidate genes is listed in Table 1.

**Figure 2:**
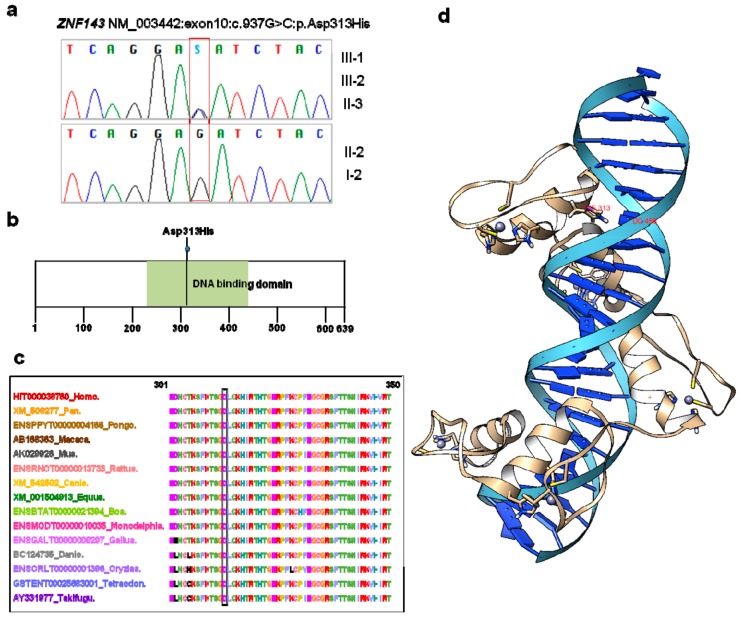
*ZNF143* mutation leading polymorphous corneal dystrophy maps to conserved domains and amino acid residues. (**a**) Sanger sequencing of the *ZNF143* gene from RNA. (**b**) ZNF143 is a 639-amino-acid protein containing a DNA-binding domain. (**c**) The ZNF143 positions affected by missense alterations (box) show amino acid conservation between species among vertebrates. (**d**) The complex structure of ZNF143 p.Asp313His and DNA is shown in a cartoon representation generated by using UCSF Chimera. A mutation residue from Asp to His is shown with the label HIS313. Zinc ions in grey are shown with two Histidine and two Cytosine residues.

Sanger sequencing of genomic DNA and RNA confirmed the presence of the heterozygous *ZNF143* c.937G>C p.(Asp313His) mutation in the three patients but not in the unaffected family members (I-2 and II-6) (Figure 2a). ZNF143 p.(Asp313His) was not present in any of the large population databases or the ethnic-specific database employed in this study. In addition, we did not find this variant in over 100 ethnically matched Korean control individuals by Sanger sequencing. The variant was determined to be located on a DNA binding domain (Figure 2b) and was predicted to be pathogenic by SIFT (deleterious 0.02), PolyPhen2 (probably damaging 0.949), and MutationTaster (disease causing). The variant was shown to affect highly conserved amino acid residues across taxa from pufferfish to humans (Figure 2c).

### 3.3. Three-Dimensional Modeling of ZNF143 p.Asp313His

We generated a three-dimensional (3D) model of ZNF143 p.Asp313His. The complex structure of ZNF143 p.Asp313His and DNA is shown in the cartoon representation of Figure 2d. The negatively charged Asp is the strongest binder for cytosine (labeled with DC458), forming a hydrogen bond [29]. The mutation from p.Asp313His weakens the binding between ZNF143 and DNA.

### 3.4. In Vitro Functional Studies of ZNF143 c.937G>C p.Asp313His

**Figure 3:**
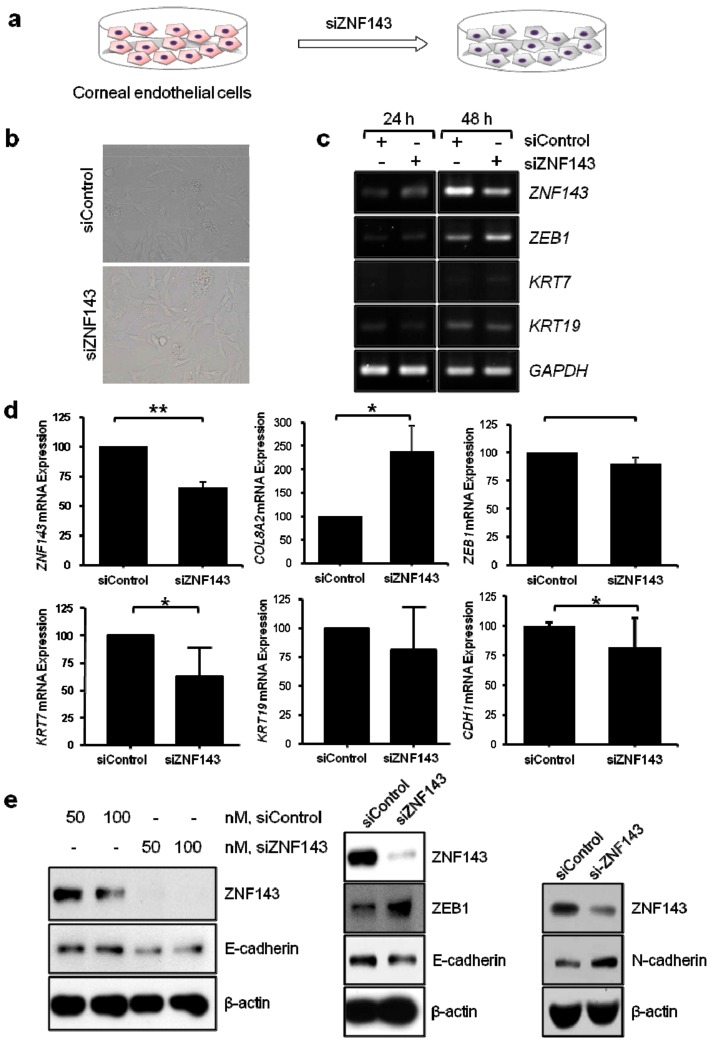
The effect of ZNF143 expression in human corneal endothelial cells (hCECs). (**a**) hCECs were transfected with small interfering (si)RNA against *ZNF143* (siZNF143) or control siRNA and grown for 24 h or 48 h. (**b**) Before harvest, pictures of the cells were taken at 20× magnification by using an inverted light microscope (Olympus, Japan). Cells were harvested for RT-PCR (**c**), RT-qPCR (**d**), and immunoblotting (**e**). siZNF143 transfected hCECs revealed a decreased expression of *KRT7*, *CDH1*, and E-cadherin, and an increased expression of *COL8A2*, N-cadherin, and *ZEB1*. The results shown are representative of at least three independent experiments.

We proceeded to perform in-vitro functional studies to identify the detailed mechanism of ZNF143 in endothelial CD development. ZNF143 knockdown (KD) was successfully undertaken by small interfering (si)RNAs, which decreased the expression of *KRT7* and E-cadherin and increased expression of *ZEB1* and N-cadherin (Figure 3).

**Figure 4:**
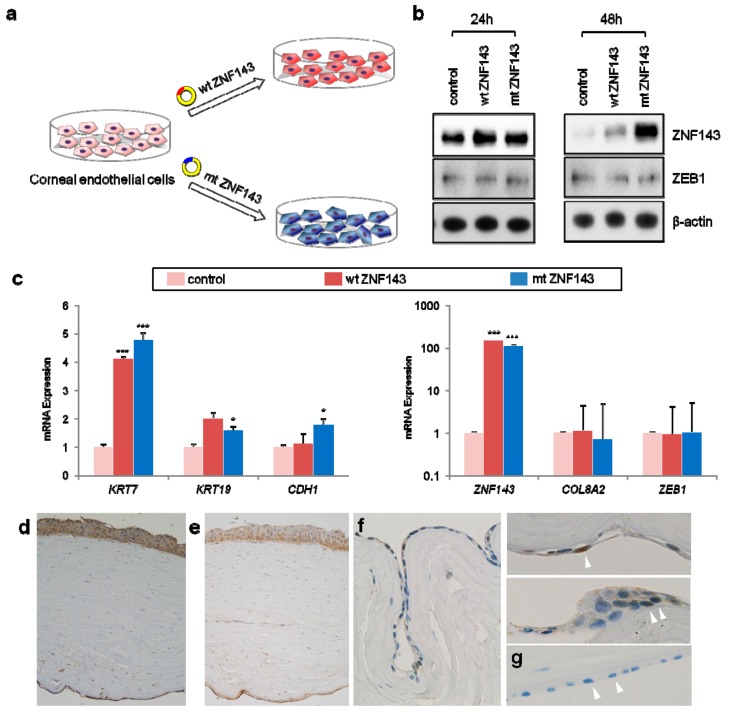
The effect of wild-type (wt) *ZNF143* and mutant *ZNF143* p.Asp313His (mt) in human corneal endothelial cells (hCECs). (**a**) A schematic diagram to investigate the role of ZNF143 in hCECs. hCECs were transfected with plasmids encoding human wt or mt *ZNF143* cDNA for 24–48 h and harvested for RT-qPCR and immunoblotting. (**b**) The expressions of ZNF143 and ZEB1 were verified by immunoblotting in hCECs transfected with wt or mt *ZNF143*. Data shown are representative of at least three independent experiments. (**c**) The effects of *ZNF143* on various genes associated with corneal dystrophy and epithelialization were analyzed in hCECs transfected with wt or mt *ZNF143* by RT-qPCR. *COL8A2* and *ZEB1* expression was not different in hCECs transfected with wt or mt *ZNF143* compared to the control. Data are plotted by mean and standard deviation. *OVOL2* is not expressed in hCECs used for this study. The expression of *KRT7*, *KRT19*, and *CDH1* increased significantly in mt *ZNF143* transfected hCECs. They reveal increased expression in wt *ZNF143*-transfected cells with or without statistical significance. * *p* < 0.05, *** *p* < 0.001. Immunohistochemical staining revealed that ZEB1 (**d**) and COL8A2 (**e**) were positively stained both in corneal epithelium and endothelium. ZNF143 was expressed in cytoplasm and nucleus (arrow heads) of patient CECs (**f**) while it was not expressed in control CECs (**g**).

Then, we transfected human hCECs with plasmids encoding wild-type (wt), mutant (mt) *ZNF143*, or an empty vector control and examined their effect on hCECs (Figure 4a). Mutant ZNF143 continued to highly express until 48 h after transfection, while wild-type ZNF143 expression declined (Figure 4b). Genes associated with epithelial cells including *KRT7*, *KRT19*, and *CDH1* were upregulated in wild-type and mutant *ZNF143*-transfected hCECs. RT-qPCR demonstrated that endothelial CD-associated gene expression did not change (Figure 4c). These changes were coherently observed in gene expression microarray data and immunohistochemical staining on patient tissue. ZEB1 and COL8A2 were expressed in the corneal epithelium and endothelium. ZNF143 stained positive in the cytoplasm and nucleus of endothelial cells (Figure 4d–f), while it did not stain in normal cornea (Figure 4g).

To ascertain the effect of mutant *ZNF143*, we transfected HCT116, HEK293 and HMEC-1 with wild-type or mutant *ZNF143*. The results bolster the role of mutant *ZNF143* in the upregulation of CK7 and E-cadherin of the HCT116 and HEK293 cells, and the downregulation of α-SMA and Snail in the HMEC-1. In HEK293 cells, the expression of wt or mt ZNF143 increased CK7 and E-cadherin proteins, regardless of a tagging FLAG on its N-terminus, and decreased ZEB1 promoter activities. The results shown are representative of at least three independent experiments (Figure S2).

### 3.5. Network Analysis Reveals Activation of Epithelialization and Proinflammatory Signals

Gene expression profiles were obtained to explore the relationships among significantly changed biomolecules. Ingenuity^®^ Pathway Analysis (IPA) on this gene set shows a network diagram illustrating annotated interactions between *ZNF143* and genes associated with PPCD. These results indicate that the altered top diseases and bio-functions included ‘epithelial cancer’ (*p* = 8.57 × 10^−14^), ‘activation of epithelial cell lines’ (*p* = 9.00 × 10^−5^), ‘dystrophy of cornea’ (*p* = 3.10 × 10^−5^), and ‘PPCD’ (*p* = 3.24 × 10^−4^). GSEA [27] reveals that 81 pathways, including ‘BROWNE_INTERFERON_ RESPONSIVE_GENES’, ‘SANA_RESPONSE_TO_IFNG_UP’, and ‘REACTOME_IMMUNE_ SYSTEM’, were positively correlated with genes in the gene set in mutant *ZNF143* transfected hCECs (Table S1, Figure S3).

We measured *ZNF143* expression in the patients’ peripheral blood leukocytes and found that the expression was higher compared to their family members and age-matched normal controls (Figure S4a). Additionally, we analyzed differently expressed genes in patients’ peripheral blood leukocytes using IPA and identified canonical pathways including the role of interleukin(IL)-17F in allergic inflammatory airway disease, lymphotoxin β receptor signaling, triggering receptor expressed on myeloid cells-1 (TREM1) signaling and IL-8 and IL-6 signaling (*p* ≤ 10^−3^, z-score ≥ 2). Network analysis shows a map of connected and overlapping canonical pathways (Figure S4b).

## 4. Discussion

In this study, we first linked a novel mutation of *ZNF143* with endothelial CD. Clinical and pathological features of three patients were compatible with endothelial CD. We did not detect any mutation of known disease-causing genes by Sanger sequencing. It became more clear that the patients’ disease was caused by a previously unknown genetic mutation after haplotype analysis which did not reveal a common shared allele associated with PPCD1 or CHED1 on the short arm of chromosome 20 [14]. Therefore, we undertook WES to find a possible candidate gene of endothelial CD in our Korean family. Through a sequential genetic approach and integrated investigations, *ZNF143* c.937G>C p.(Asp313His) represented the best candidate for the cause of our patients’ endothelial CD.

ZNF143 is a sequence-specific DNA-binding protein encoded by the *ZNF143* gene located on chromosome 11p15.4. The protein is directly recruited to the promoter of genes engaged in chromatin interactions, where it binds to its DNA recognition sequence [30,31]. The protein segment ranging from residue 237 to 440 contains seven zinc-fingers each consisting of an alpha helix and an anti-parallel beta sheet. The 3D model supported the proposal that weakened binding between mutant ZNF143 and DNA can be associated with disease pathogenesis.

The following in-vitro functional studies supported the detailed mechanism of ZNF143 in endothelial CD development. Preferentially, we knocked down *ZNF143* in cultured hCECs and evaluated gene expression changes. The absence of overt PPCD-like changes in *ZNF143* KD hCECs suggested that heterozygous *ZNF143* p.Asp313His in our patients is not a loss-of-function mutation. This reasoning is supported by the description of patients with a homozygous deletion of 11p14-15 and since the compound heterozygous *ZNF143* mutation did not show corneal involvement [32,33,34]. Therefore, we performed a transfection study. ZNF143 expression was upregulated in the patients’ corneal endothelium and peripheral blood as well as mutant-transfected hCECs. The epithelialization of corneal endothelium was not only demonstrated by the immunohistochemical staining of patient tissue, but also verified in mutant-transfected hCECs. We carefully postulate that mutant *ZNF143* plays an important role in the reverse epithelial-to-mesenchymal transition [35], resulting in the epithelialization of the corneal endothelium. The following experiment showed that mutant *ZNF143* potentiated the expression of epithelial markers and decreased the expression of mesenchymal markers, combined with the downregulation of snail. These findings assisted in clarifying the role of mutant *ZNF143* in the development of endothelial CD through the reverse epithelial-to-mesenchymal transition/endothelial-to- mesenchymal transition [36].

Genes associated with proinflammatory cytokine and inflammatory response were significantly enriched in mutant *ZNF143* transfected hCECs. In addition, canonical pathways associated with interleukins and inflammation were enriched in patient’s blood leukocytes. Our patients underwent visual impairment at an early age secondary to corneal edema, and all needed corneal transplantation. Even after transplantation, corneal edema and neovascularization developed on account of immune response. These results told us that genes working on the regulation of proinflammatory cytokine responsiveness were commonly activated in cells with *ZNF143* p.Asp313His.

## 5. Conclusions

In conclusion, we have identified a missense mutation in the DNA-binding domain of *ZNF143* as a best candidate for endothelial CD. The mutation seems to result in the epithelial-like metaplasia of corneal endothelium with stromal edema. We anticipate that more endothelial CD linked by *ZNF143* mutations will be discovered once *ZNF143* is recognized as a causal gene. Further study is needed clarify the role of *ZNF143* in endothelial CD.

## Figures and Tables

**Table 1:** Summary of evidence for the pathogenicity classification of candidate rare heterozygous variants identified by whole exome sequencing.

Chr	Gene	Annotation	SIFT (Score)	PolyPhen (Score)	Mutation Taster	Evola	dbSNP	GnomAD Exomes	KRG DB	ACMG Criteria	ACMG Classification
1	*TXNIP*	NM_006472:exon5: c.A805G:p.I269V	deleterious 0.04	probably damaging 0.996	disease causing	conserved (12/12)	-	0.000003977	-	BS2, PP3, PP4	VUS
2	*EPC2*	NM_015630:exon8: c.G1208A:p.R403Q	deleterious 0.01	benign 0.383	disease causing	conserved (11/15)	rs761809638	0.00008181	-	BS2, PP4	VUS
9	*KCNV2*	NM_133497:exon1: c.G217C:p.E73Q	tolerated 0.08	benign 0.005	polymorphism	conserved (12/13)	rs752013234	0.00001593	-	BS2, PP4	VUS
9	*NUTM2F*	NM_017561:exon7: c.C1766G:p.A589G	deleterious 0.05	probably damaging 0.985	polymorphism	no data	rs201719890	0.04351	0.2363	BA1, PP4	Benign (I)
9	*AOPEP*	NM_001193329: exon10:c.A1960G:p.S654G		benign 0.018	polymorphism	conserved (7/15)	-	-	-	PM2, PP4	VUS
9	*TNC*	NM_002160:exon3: c.C1063T:p.R355W	deleterious 0	possibly damaging 0.677	disease causing	conserved (8/15)	rs779621288	0.00001195	-	BS2, PP3, PP4	VUS
9	*RABGAP1*	NM_012197:exon10: c.G1221A:p.M407I	tolerated 0.07	benign 0.015	disease causing	N/A	rs769879519	0.000008117	-	BS2, PP4	VUS
11	*ZNF143*	NM_003442:exon10: c.G937C:p.D313H	deleterious 0.02	probably damaging 0.949	disease causing	conserved (15/15)	-	-	-	PM2, PP3, PP4	Likely pathogenic (II)
13	*ERICH6B*	NM_182542:exon3: c.C302A:p.A101E	Tolerated 1	unknown	polymorphism	no data	-	-	-	PM2, PP4	VUS
19	*MZF1*	NM_003422:exon6: c.G1952A:p.R651Q	deleterious 0.04	probably damaging 0.994	polymorphism	conserved (9/9)	rs201221836	0.0002058	0.0105	BS2, PP4	VUS

Chr, chromosome; DB, database; GnomAD, genome aggregation database; KRG, Korean Reference Genome; ACMG, American College of Medical Genetics.

## Supplementary Materials

The following are available online at https://www.mdpi.com/2077-0383/8/8/1174/s1, Figure S1: Genetic evaluation of posterior polymorphous corneal dystrophy; Figure S2: The effect of wild-type (wt) and mutant (mt) *ZNF143* in HCT116 or HEK293 cells; Figure S3: Network analysis of gene expression; Figure S4: Gene expression in patient’s peripheral blood; Table S1: Summary of gene set enrichment analysis (GSEA) in human corneal endothelial cells transfected with ZNF143 p.Asp313His; List of all URL in the manuscript.

## References

[1] Weiss J.S., Moller H.U., Aldave A.J., Seitz B., Bredrup C., Kivela T., Munier F.L., Rapuano C.J., Nischal K.K., Kim E.K. (2015). IC3D classification of corneal dystrophies—Edition 2. Cornea.

[2] Vemuganti G.K., Rathi V.M., Murthy S.I. (2011). Histological landmarks in corneal dystrophy: Pathology of corneal dystrophies. Dev. Ophthalmol..

[3] Klintworth G.K. (2009). Corneal dystrophies. Orphanet J. Rare Dis..

[4] Chae H., Kim M., Kim Y., Kim J., Kwon A., Choi H., Park J., Jang W., Lee Y.S., Park S.H. (2016). Mutational spectrum of Korean patients with corneal dystrophy. Clin. Genet..

[5] Schmedt T., Silva M.M., Ziaei A., Jurkunas U. (2012). Molecular bases of corneal endothelial dystrophies. Exp. Eye Res..

[6] Biswas S., Munier F.L., Yardley J., Hart-Holden N., Perveen R., Cousin P., Sutphin J.E., Noble B., Batterbury M., Kielty C. (2001). Missense mutations in COL8A2, the gene encoding the alpha2 chain of type VIII collagen, cause two forms of corneal endothelial dystrophy. Hum. Mol. Genet..

[7] Krafchak C.M., Pawar H., Moroi S.E., Sugar A., Lichter P.R., Mackey D.A., Mian S., Nairus T., Elner V., Schteingart M.T. (2005). Mutations in TCF8 cause posterior polymorphous corneal dystrophy and ectopic expression of COL4A3 by corneal endothelial cells. Am. J. Hum. Genet..

[8] Aldave A.J., Han J., Frausto R.F. (2013). Genetics of the corneal endothelial dystrophies: An evidence-based review. Clin. Genet..

[9] Liskova P., Dudakova L., Evans C.J., Rojas Lopez K.E., Pontikos N., Athanasiou D., Jama H., Sach J., Skalicka P., Stranecky V. (2018). Ectopic GRHL2 Expression Due to Non-coding Mutations Promotes Cell State Transition and Causes Posterior Polymorphous Corneal Dystrophy 4. Am. J. Hum. Genet..

[10] Kirkness C.M., McCartney A., Rice N.S., Garner A., Steele A.D. (1987). Congenital hereditary corneal oedema of Maumenee: Its clinical features, management, and pathology. Br. J. Ophthalmol..

[11] Davidson A.E., Liskova P., Evans C.J., Dudakova L., Nosková L., Pontikos N., Hartmannová H., Hodaňová K., Stránecký V., Kozmík Z. (2016). Autosomal-Dominant Corneal Endothelial Dystrophies CHED1 and PPCD1 Are Allelic Disorders Caused by Non-coding Mutations in the Promoter of OVOL2. Am. J. Hum. Genet..

[12] Vithana E.N., Morgan P., Sundaresan P., Ebenezer N.D., Tan D.T., Mohamed M.D., Anand S., Khine K.O., Venkataraman D., Yong V.H. (2006). Mutations in sodium-borate cotransporter SLC4A11 cause recessive congenital hereditary endothelial dystrophy (CHED2). Nat. Genet..

[13] Kumar A., Bhattacharjee S., Prakash D.R., Sadanand C.S. (2007). Genetic analysis of two Indian families affected with congenital hereditary endothelial dystrophy: Two novel mutations in SLC4A11. Mol. Vis..

[14] Heon E., Mathers W.D., Alward W.L., Weisenthal R.W., Sunden S.L., Fishbaugh J.A., Taylor C.M., Krachmer J.H., Sheffield V.C., Stone E.M. (1995). Linkage of posterior polymorphous corneal dystrophy to 20q11. Hum. Mol. Genet..

[15] Aldave A.J., Yellore V.S., Vo R.C., Kamal K.M., Rayner S.A., Plaisier C.L., Chen M.C., Damani M.R., Pham M.N., Gorin M.B. (2009). Exclusion of positional candidate gene coding region mutations in the common posterior polymorphous corneal dystrophy 1 candidate gene interval. Cornea.

[16] Richards S., Aziz N., Bale S., Bick D., Das S., Gastier-Foster J., Grody W.W., Hegde M., Lyon E., Spector E. (2015). Standards and guidelines for the interpretation of sequence variants: A joint consensus recommendation of the American College of Medical Genetics and Genomics and the Association for Molecular Pathology. Genet. Med. Off. J. Am. Coll. Med Genet..

[17] Joo K., Joung I., Lee S.Y., Kim J.Y., Cheng Q., Manavalan B., Joung J.Y., Heo S., Lee J., Nam M. (2016). Template based protein structure modeling by global optimization in CASP11. Proteins.

[18] Modi V., Xu Q., Adhikari S., Dunbrack R.L. (2016). Assessment of template-based modeling of protein structure in CASP11. Proteins.

[19] Joo K., Lee J., Kim I., Lee S.J., Lee J. (2008). Multiple sequence alignment by conformational space annealing. Biophys. J..

[20] Joo K., Lee J., Seo J.H., Lee K., Kim B.G., Lee J. (2009). All-atom chain-building by optimizing MODELLER energy function using conformational space annealing. Proteins.

[21] Lee J., Scheraga H.A., Rackovsky S. (1997). New optimization method for conformational energy calculations on polypeptides: Conformational space annealing. J. Comput. Chem..

[22] Weiner P.K., Kollman P.A. (1981). AMBER: Assisted model building with energy refinement. A general program for modeling molecules and their interactions. J. Comput. Chem..

[23] Peters M.B., Yang Y., Wang B., Füsti-Molnár L., Weaver M.N., Merz K.M. (2010). Structural Survey of Zinc Containing Proteins and the Development of the Zinc AMBER Force Field (ZAFF). J. Chem. Theory Comput..

[24] Pettersen E.F., Goddard T.D., Huang C.C., Couch G.S., Greenblatt D.M., Meng E.C., Ferrin T.E. (2004). UCSF Chimera—A visualization system for exploratory research and analysis. J. Comput. Chem..

[25] Paek A.R., Lee C.H., You H.J. (2014). A role of zinc-finger protein 143 for cancer cell migration and invasion through ZEB1 and E-cadherin in colon cancer cells. Mol. Carcinog..

[26] Kramer A., Green J., Pollard J., Tugendreich S. (2014). Causal analysis approaches in Ingenuity Pathway Analysis. Bioinformatics.

[27] Subramanian A., Tamayo P., Mootha V.K., Mukherjee S., Ebert B.L., Gillette M.A., Paulovich A., Pomeroy S.L., Golub T.R., Lander E.S. (2005). Gene set enrichment analysis: A knowledge-based approach for interpreting genome-wide expression profiles. Proc. Natl. Acad. Sci. USA.

[28] Goh G., Choi M. (2012). Application of whole exome sequencing to identify disease-causing variants in inherited human diseases. Genom. Inform..

[29] De Ruiter A., Zagrovic B. (2015). Absolute binding-free energies between standard RNA/DNA nucleobases and amino-acid sidechain analogs in different environments. Nucleic Acids Res..

[30] Myslinski E., Krol A., Carbon P. (1998). ZNF76 and ZNF143 are two human homologs of the transcriptional activator Staf. J. Biol. Chem..

[31] Bailey S.D., Zhang X., Desai K., Aid M., Corradin O., Cowper-Sal Lari R., Akhtar-Zaidi B., Scacheri P.C., Haibe-Kains B., Lupien M. (2015). ZNF143 provides sequence specificity to secure chromatin interactions at gene promoters. Nat. Commun..

[32] Bitner-Glindzicz M., Lindley K.J., Rutland P., Blaydon D., Smith V.V., Milla P.J., Hussain K., Furth-Lavi J., Cosgrove K.E., Shepherd R.M. (2000). A recessive contiguous gene deletion causing infantile hyperinsulinism, enteropathy and deafness identifies the Usher type 1C gene. Nat. Genet..

[33] Pupavac M., Watkins D., Petrella F., Fahiminiya S., Janer A., Cheung W., Gingras A.C., Pastinen T., Muenzer J., Majewski J. (2016). Inborn Error of Cobalamin Metabolism Associated with the Intracellular Accumulation of Transcobalamin-Bound Cobalamin and Mutations in ZNF143, Which Codes for a Transcriptional Activator. Hum. Mutat..

[34] Halbig K.M., Lekven A.C., Kunkel G.R. (2012). The transcriptional activator ZNF143 is essential for normal development in zebrafish. BMC Mol. Biol..

[35] Kawatsu Y., Kitada S., Uramoto H., Zhi L., Takeda T., Kimura T., Horie S., Tanaka F., Sasaguri Y., Izumi H. (2014). The combination of strong expression of ZNF143 and high MIB-1 labelling index independently predicts shorter disease-specific survival in lung adenocarcinoma. Br. J. Cancer.

[36] Ghosh A.K., Nagpal V., Covington J.W., Michaels M.A., Vaughan D.E. (2012). Molecular basis of cardiac endothelial-to-mesenchymal transition (EndMT): Differential expression of microRNAs during EndMT. Cell. Signal..

